# Evaluation of efficiency and safety of oral corticosteroid therapy in children patients with exacerbations of asthma: A protocol for systematic review and meta-analysis

**DOI:** 10.1097/MD.0000000000026250

**Published:** 2021-06-18

**Authors:** Zuowu Chen, Lei Zhang, Jinbing You, Jiangjiang Wang, Guilan Chen

**Affiliations:** a Department of Pediatrics, the First People's Hospital of Jiangxia District; b Department of Pediatrics, Hubei Maternal and Child Health Care Hospital, Wuhan, Hubei, PR China.

**Keywords:** asthma, corticosteroid, efficacy, exacerbations, meta

## Abstract

**Background:**

Asthma is the most frequently occurring obstructive airway disease, it inflicts the highest morbidity among children. Among the paediatric populace, severe exacerbations of asthma are a common reason behind patient consultations and hospitalizations. Oral corticosteroids are a primary component in the treatment of asthma exacerbations; however, there is controversy regarding how corticosteroids functions.

**Methods:**

The present review will conduct a search on MEDLINE, EMBASE, Cochrane Library, China National Knowledge Infrastructure, and Chinese BioMedical Literature. The search will cover the databases from their beginning to May 2021. The search aims to identify all the randomized controlled studies on oral corticosteroids in treating children with asthma exacerbations. Two independent authors will choose studies, perform data extraction, and use an appropriate tool to assess the bias risk in the selected articles. Moreover, a sensitivity analysis will be performed to assess the robustness of the results. The RevMan (version 5.3) software will be employed to perform data synthesis and statistical analysis.

**Results:**

This study will examine the efficiency and safeness of oral corticosteroid therapy to treat children with asthma exacerbations by pooling the results of individual studies.

**Conclusion:**

The findings of this study will provide vigorous evidence to judge whether oral corticosteroid therapy is an efficiency strategy to treat patients with asthmatic exacerbations.

**OSF registration number:**

May 20, 2021.osf.io/3ghjt. (https://osf.io/3ghjt/).

## 1 Introduction

Childhood asthma refers to a lasting inflammatory condition of the airways. It is mainly characterized by episodic and rescindable airway constriction and inflammation occurring as a response to environmental allergens, an infection, and irritants.^[1]^ Acute exacerbation of asthma is a leading cause of admissions to the emergency department, and children are more susceptible to chronic pulmonary impairments.^[2–4]^ Even though present asthma control indicates prospective exacerbations, asthmatic exacerbations are likely to occur even in those having healthy lung functionality.^[5,6]^ Asthma exacerbation treatment relies on the exacerbation severity. The primary form of treatment to manage acute asthmatic exacerbations entails titrated oxygen delivery, systemic corticosteroid treatment, administration of intermittent inhaled short-acting beta2-agonists, and ipratropium bromide.^[7–9]^ Regardless of advanced therapeutic measures and guideline-based care, asthma poses a substantial strain on the public health sector.

Systemic corticosteroids refer to powerful anti-inflammatory agents for treating asthma, which can reduce hospitalizations and enhance the functionality of the lungs in asthmatic patients.^[10,11]^ Oral corticosteroids are suggested to treat all asthma exacerbations apart from the most mild cases; they require prompt initiation.^[7,11]^ The effectiveness of systemic corticosteroids is well-established, and widely acknowledged by clinicians. However, the benefit of oral corticosteroids to patients is yet to be clearly established. Presently, oral corticosteroids are approved for children with exacerbations of asthma. Therefore, it is critical to comprehend the effectiveness and safeness of using oral corticosteroids therapy to treat children with asthmatic exacerbations. Therefore, the present study aims to assess the efficacy and safeness of adopting oral corticosteroids therapy to treat children with asthmatic exacerbations.

## 2 Objectives

To investigate the efficacy and safeness of oral corticosteroids therapy to treat children with asthma exacerbations.

## 3 Methods

### 3.1 Study registration

The current protocol has been registered in OSF (https://osf.io/) under number 10.17605/OSF.IO/3GHJT. This protocol report is conducted as per the guidelines defined by Preferred Reporting Items for Systematic review and Meta-Analysis Protocol (PRISMA-P) statement.^[12]^

### 3.2 Eligibility criteria for including studies

#### 3.2.1 Types of studies

The present protocol report will consist of all Randomized Control Trials that assessed the efficacy and level of safety when using oral corticosteroids therapy to treat asthmatic children. The language of the studies will be limited to English and Chinese.

#### 3.2.2 Types of participants

The study will include children (any person younger than 18 years of age was defined as a child) with exacerbations of asthma diagnosed by a medical practitioner.

#### 3.2.3 Types of interventions/comparisons

The present study will include research that compared oral corticosteroids with placebo or other normal care, when standard care did not include corticosteroids. Moreover, this protocol report will also include researches that utilized oral corticosteroids with any dose or treatment duration.

#### 3.2.4 Types of outcome measures

The major outcomes for the present study include hospitalizations, asthmatic symptoms once the corticosteroids course concludes, and adverse outcomes. The minor outcomes for the present study include fresh exacerbations in the posttreatment analysis period, lung functionality tests when treatment concludes, all side effects, and all adverse outcomes.

### 3.3 Search methods for identification of studies

The present review will conduct a search on electronic databases, including MEDLINE, EMBASE, Cochrane Library, China National Knowledge Infrastructure, and Chinese BioMedical Literature. The search will cover the databases from their beginning to May 2021 to identify all the randomized controlled studies on oral corticosteroids in treating children with exacerbations of asthma. The search terms listed below will either be utilised alone or in combination: “asthma,” “corticosteroids,” and “randomized controlled trial.” Moreover, we will also go through all reference lists in the included primary studies and review articles to recognize all related studies.

### 3.4 Data collection and analysis

#### 3.4.1 Selection of studies

**Figure 1:**
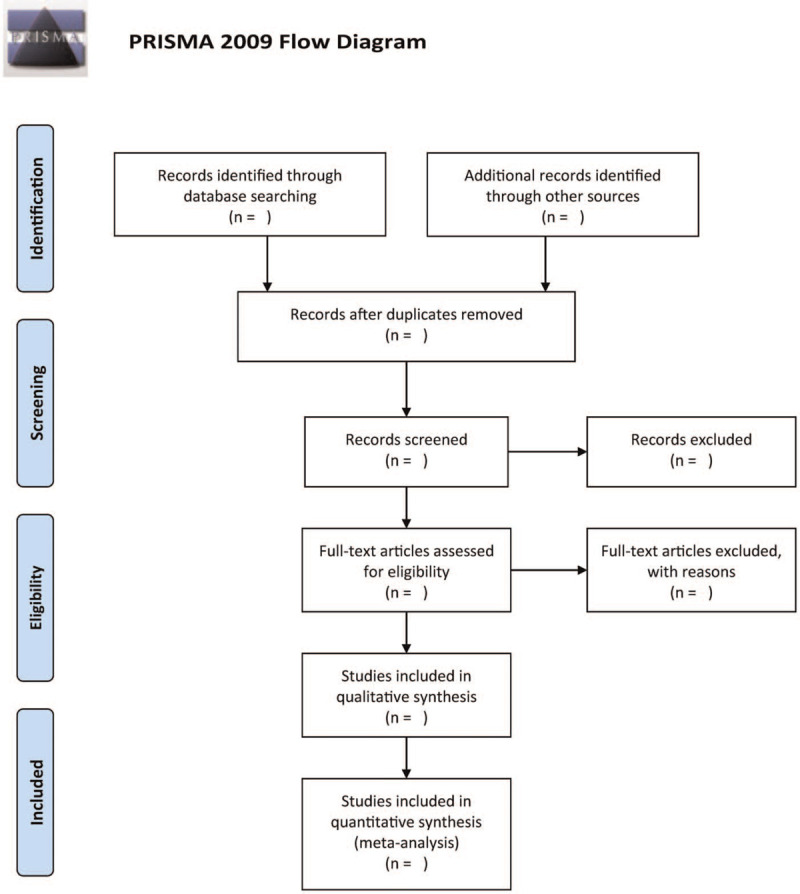
The research flowchart.

The retrieved studies will be screened by 2 independent authors. The authors will read the title/abstract to identify and remove duplicate research articles as well as studies that do not match the inclusion criteria. The authors will scrutinize the full text in all the identified studies to select the ones that satisfy the inclusion criteria. All disagreements will be resolved via discussions with a third independent author. Figure 1 shows the complete study selection process.

#### 3.4.2 Data extraction

Three authors will design a pilot data extraction form. Two autonomous authors will independently extract data via the final data extraction form. All disagreements will be resolved via discussion with a third independent author. The data extraction content includes the following: basic data (first author, published year, ethnicity, nationality, age, and gender), design of study (number of participants, randomization, binding methods, allocation concealment, intervention method, and treatment duration), and outcome measures.

#### 3.4.3 Assessment of risk of bias

Based on the Cochrane Collaboration tool 2 autonomous authors will determine the bias risk of the included studies.^[13]^ All disagreements will be resolved through discussions with a third author.

#### 3.4.4 Measures of treatment effect

The mean differences or standardized mean differences (SMDs) with 95% confidence intervals will be utilized for continuous outcome data. Moreover, the relative risk with 95% confidence interval will be utilized for dichotomous outcome data.

#### 3.4.5 Dealing with missing data

It is planned to contact investigators to authenticate critical research characteristics and if applicable, acquire lost numerical outcomes of data.

#### 3.4.6 Assessment of heterogeneity

This study will employ the standard *X*^*2*^ test and *I*^*2*^ statistic to evaluate the heterogeneity. In the event where substantial heterogeneity is identified (*P* < .1, and *I*^*2*^ > 50%), the random-effects models will be adopted; otherwise, the fixed-effects model will be utilized.^[14,15]^

#### 3.4.7 Assessment of reporting biases

This study will make use of funnel plots to investigate any potential small study and publication bias when there are over ten studies.

#### 3.4.8 Sensitivity analysis

We will perform sensitivity analysis to assess how using random assumptions of data could influence the robustness and reliability of the collective results.

## 4 Discussion

This protocol study aims to assess the efficacy and level of safety when using oral corticosteroids therapy to treat children with asthmatic exacerbations. Recently, there has been a gradual increment in the RCTs studying oral corticosteroids therapy for treating asthmatic children. Admittedly, numerous published studies have suggested that the application of oral corticosteroids holds a significant position for treating children with asthmatic exacerbations. However, the efficacy and safety level of oral corticosteroids therapy when used to treat children with asthmatic exacerbations is yet to be established. Therefore, this study will assess the effectiveness and security of oral corticosteroids therapy to treat asthmatic children. It is hoped that this review will provide vigorous evidence to outline the efficacy and safeness of using oral corticosteroids therapy to treat asthmatic children. The outcomes which be of great use for medical practitioners, scholars, and patients.

## Author contributions

**Conceptualization:** Zuowu Chen, Guilan Chen.

**Data curation:** Zuowu Chen, Lei Zhang, Guilan Chen.

**Formal analysis:** Zuowu Chen, Jiangjiang Wang.

**Investigation:** Lei Zhang, Jiangjiang Wang.

**Project administration:** Zuowu Chen, Lei Zhang, Jiangjiang Wang, Guilan Chen.

**Resources:** Jiangjiang Wang.

**Software:** Lei Zhang, Jiangjiang Wang.

**Supervision:** Lei Zhang.

**Validation:** Lei Zhang, Jiangjiang Wang.

**Visualization:** Jinbing You, Guilan Chen.

**Writing – original draft:** Zuowu Chen, Guilan Chen.

**Writing – review & editing:** Jinbing You, Guilan Chen.
